# On the Anatomy and Diseases of the Urinary and Sexual Organs; Containing the Anatomy of the Bladder and Urethra, and the Treatment of the Obstructions to Which These Passages Are Liable

**Published:** 1844-01

**Authors:** 


### ART. XIV.

1. *On the Anatomy and Diseases of the Urinary and Sexual Organs: containing the Anatomy of the Bladder and Urethra, and the Treatment of the Obstructions to which these passages are liable.* By G. J. GUTHRIE, F.R.S. &c. &c. Third Edition.—*London,* 1843. 8vo, pp. 156.

2. *Traité Pratique sur les Maladies des Organes Génito-urinaires.* Par le Docteur CIVIALE. Première Partie: *Maladies de l'Urètre.* Seconde Edition, considérablement augmentée.—*Paris,* 1842. 8vo, pp. 588.

*Practical Treatise on the Diseases of the Genito-urinary Organs.* By Dr. CIVIALE. Part First: *Diseases of the Urethra.* Second Edition. —*Paris,* 1842.

M. CIVIALE and Mr. Guthrie are men of great experience and great ingenuity; and anything coming from their pens is entitled to attentive consideration. But as Mr. Guthrie's work has reached a third, and M. Civiale's a second, edition, they would not under ordinary circumstances claim at our hands any lengthened notice. Nor is it our intention to give a general review of the volumes before us; but as we have reason to believe that a good deal of misapprehension exists on one important practical subject treated in them, we purpose devoting a portion of our pages to its consideration. What we refer to is the treatment of stricture.

Why is it that we have so many works on urethral diseases? It is not that they are more frequent than others; it is not that they are more difficult to treat than many ; it is not that any superior plans of treatment are suggested;—for what has the last century done to improve their treatment? Is it, sometimes at least, to constitute a vehicle for advertisement? We hope not; and yet no books are so frequently paraded in the daily papers as those treating of this class of diseases. Whatever may be the reason, we could wish that respectable men would eschew this least-to-be-desired mode of acquiring popularity. We do not, however, conceive that our continental brethren are at all less open to remark in this respect than ourselves. It is true their plan of puffing is a little different. With us it is a formal advertisement for the nonce, unblushingly paid for: with them, a man is apt to purchase an interest in one of the daily or weekly papers, and thus more insidiously, though not a whit more respectably, keeps his name and his deeds before the public.

Some idea of the scope of these works may be obtained from the fact, that Mr. Guthrie disposes, "1st, of the structure of the bladder, and, 2d, the urethra; 3d, the formation of spasmodic and permanent stricture; 4th, symptoms of and means of cure of stricture; 5th, of the treatment of impassable stricture; 6th, suppression and retention of urine; 7th, irritation of the membranous and prostatic parts of the urethra;" in 155 pages! In justice, however, to the author we should say, that he has divided the general subject into two parts, of which the present is the first; and he has done this because it is inconvenient to him to superintend the printing of a large book: and that the second part will contain "Chronic complaints of the prostate, the diseases of the bladder, the treatment of calculous affections, and the various modes of operating for the removal of a stone from the bladder."

We would respectfully suggest to Mr. Guthrie that his present title would induce us to expect more than we are likely to get. Surely the kidney is a urinary organ, and the testicle a sexual one; but nothing is said or promised of them. So is gonorrhea a disease of the urethra, but it finds no place in this work. M. Civiale takes a different course: he gives us a volume of about 600 pages on the diseases of the urethra, as much on the diseases of the prostate, and not less on those of the bladder.

Both Mr. Guthrie and M. Civiale occupy a considerable space with the anatomy of the parts concerned; of Mr. Guthrie's work more than one fourth is thus disposed of. Is this necessary? Does not every work on anatomy enter at sufficient length on the subject? Sir B. Brodie clearly thought so, for he does not devote a single page to the matter. But then it may be said, Mr. Guthrie discovered some muscular fibre which Wilson did not; and also that Mr. Wilson's ideas of the use of others were incorrect. He has also shown that the female has a prostate, which Wilson does not appear to have known; and therefore he has reasons for entering at length into the anatomy of the excretory urinary organs. And M. Civiale will no doubt say that it was important to show that the English did not know the direction of the urethra; and that, being ignorant of this point, their instruments were so formed as to facilitate the making of false passages. Mr. Guthrie in his 155 pages, and M. Civiale in his 600 pages, have confined themselves, in so far as concerns disease, almost entirely to stricture of the urethra.

First, let us see what a stricture is, and how it is produced. Both M. Civiale and Mr. Guthrie maintain that there may be a state of simple spasm of the urethra, unaccompanied by any structural change, and constituting what is known as *spasmodic* stricture. The former author allows that such a condition is most frequently developed in the membranous part of the urethra,—that portion of the canal on which the muscles of the perineum act; though it may occur in the spongy region of the urethra. For ourselves, though we would not absolutely deny the possibility of spasmodic stricture in *the absence of any irritation* or *struc-*

*tural change* at the part affected, yet we very much doubt even its occasional existence.

A *permanent* stricture, according to Mr. Guthrie, depends upon some positive alteration of structure of the wall of the canal, which causes it to thicken, and at the same time deprives it of its capability of being dilated with the same facility and to the same extent as in health. This alteration of structure is produced by inflammation, although it is difficult to account by it alone for the various appearances which these altered parts assume. A permanent stricture, according to M. Civiale, is a morbid condition of the urethral parietes, which has for effect to diminish progressively its extensibility, and at last to constitute a more or less considerable obstacle to the passage of urine. Such an obstacle may be occasioned by a kind of *bridle*, as pointed out by Sir C. Bell; by *excrescences*, by *adhesions*, and by *indurations*. No practical surgeon would deny the occasional existence of the bridle or valve-like stricture; and the evidence of Morgagni, Petit, Soemmering, Bell, Leroy, and Civiale is equally conclusive as to the possibility of the obstacle being caused by an *excrescence*; but if by *adhesion* be meant the two sides of the urethra entering into union,—we think we have no proof of such a circumstance. Undoubtedly the ordinary stricture of the urethra is caused by inflammatory action, usually of a chronic kind; set up at some point of the urethra, by which the parts become thickened and inelastic, and the passage is narrowed.

It has long been a settled point, that in probably forty-nine cases out of every fifty a stricture of the urethra is produced by a thickening of the submucous tissue; that thickening is the result of inflammation, and that inflammation is a consequence, in probably ninety-nine out of every hundred cases, of gonorrhea. And this we maintain, though M. Civiale states that a great many cases of stricture are caused by the introduction of instruments for the purpose of curing a stricture which was previously (but we conclude erroneously) supposed to exist. M. Civiale very naïvely suggests that those things are much commoner in England than in France; and further states, that the most eminent surgeons in England do not hesitate to place it in the first line among the causes of stricture. We do not refuse to admit that many men have been treated for stricture when no stricture existed, but it would be necessary to tax our credulity very largely to acknowledge that this was *a very common cause* of stricture. We have no doubt that when stricture already exists, it has often been made worse by improper treatment. But when M. Civiale states that *very distinguished* English surgeons have placed this in the first line of causes of this disease, he should have told us who they were.

Much labour has been expended in the attempt to fix the exact depth of strictures from the external orifice of the canal, but this seems to us an idle waste of time. It has been amply proved that the region of the curvature is the ordinary seat of the disease, but the distance of that region from the orifice must depend upon the development of the penis itself. M. Civiale felicitates himself on having discovered that the curators of the museums in London are all wrong in the statements appended to the preparations of stricture, and that what they describe as strictures of the membranous part of the urethra are nothing of the sort, and that they have mistaken the spongy for the membranous portion of the canal. We seem to be sadly in arrear of our French brethren in our anatomical knowledge. He thinks, also, (p. 148,) that this peculiarity was a source of mistakes in the application of the proper method of treatment. We admit that M. Civiale is well qualified to decide upon such a point, but we are really so ignorant as not to know that a stricture at five inches requires a different plan of treatment to one situated a quarter of an inch further from the orifice.

Although the ordinary seat of stricture be that of the curvature, yet a contraction may occur at any point from the orifice of the urethra to the prostate.

M. Civiale thinks that the morbid structure constituting the obstruction varies with the region affected, and that the treatment should vary with the region. We have carefully examined a large number of specimens of stricture in the different regions of the canal, but we are not sensible of any other difference than is caused by the greater or less density, which is probably dependent upon its duration,—except in some cases in the neigbourhood of the orifice, where the obstruction is caused by the cicatrization of an ulcer.

Nothing is more simple, under ordinary circumstances, than to ascertain the existence of a stricture, provided the surgeon possess moderate dexterity. At the same time, a bungler is frequently deceived, and patients are subjected to treatment when no proper obstacle exists.

A stricture rarely exists long without being accompanied by a discharge,—it may not be constant, but it will return again and again,—and by a difficulty in passing the urine; it may be, also, by disturbance in the bladder or the kidney. No prudent surgeon, however, will pronounce a confident opinion upon the existence of a stricture until he has carefully examined the canal. When, by the introduction of a common bougie, the surgeon has ascertained that a stricture exists, but has failed to penetrate into it, Mr. Guthrie gives the following directions as to the proper mode of proceeding:

"It will be necessary to take an impression of the face of the stricture, in order to discover where the opening is situated. This is accomplished by means of a model bougie, the point of which is made of softer materials than the shaft. The point having been made sufficiently soft by heat, should be well oiled, and passed down to the stricture, against the face of which it is to be gently but steadily pressed. If the stricture, although narrow, is not very tough or permanent, it will sometimes yield, and the soft bougie will go through, when the point of a harder one only bends, twists, or turns back, the bougie having doubled on itself in the passage. If the stricture should not yield, the point of the model bougie does, and is gradually pressed into the sinuosities or openings on its surface, so that after remaining some two or three minutes, it may be withdrawn with one or more processes or marks projecting from the end of it, either acute or obtuse, as the case may be, and indicating the commencement of the true, and sometimes also that of one or more false passages." (p. 72.)

Now we will venture to say that no more fallacious guide could be called in to our assistance in such cases than the model bougie,—and we may as well quote M. Civiale in confirmation of our opinion.

"As a means of diagnosis," says he, "the *sonde exploratrice* has been frequently substituted for the common bougie. As the advantages of this instrument have been much exaggerated, it is important to ascertain its true value. The *sonde exploratrice* does not always produce the happy effects which have been attributed to it, and very frequently it is a faithless guide. At the same time that it will sometimes give us an useful indication, we must not lose sight of the fact that it will frequently deceive us, especially when the part to be modelled is at the curvature, and that is the usual seat of stricture. There, instead of penetrating into the contracted point, it is pressed almost into a globe, which, in its withdrawal, is apt to irritate the remainder of the canal. We have even seen cases in which a simple spasmodic contraction was sufficient to prevent it from penetrating, so as to give the idea of organic stricture, when a sound of large size would pass easily. And even when, under favorable circumstances, the soft bougie penetrates into the contraction, so as to make known the size, and, it may be, the position of the opening, experience shows that the evidence cannot always be relied on. If there be any spasm, the bougie indicates a smaller opening than the actual one. This is shown, by comparing the size of the model with that of a bougie which can be passed immediately after. And, whatever may be said to the contrary, the model rarely gives an exact indication of the position of the orifice, because all points of the circumference of the stricture do not offer an equal resistance." (pp. 173-5.)

This uncertainty has been proved by daily observation, and we can only wonder that a man of Mr. Guthrie's experience should advocate the use of an instrument so faithless in most cases, and the use of which is not unattended with pain.

As to the important question, how should stricture be treated ? we find little—shall we say nothing ?—that is new in either of the works before us. In the employment of dilatation both concur; and as to the result of dilatation there is little difference of opinion. Mr. Guthrie says, "a stricture cannot be cured by dilatation until such time as a passage has been obtained through it sufficient to admit a small bougie." The instrument must be gradually increased, until one "as large as the orifice will admit will at last proceed through the whole passage without meeting with any obstacle; and it ought to be repeated at longer intervals until the disposition for contraction seems to be removed, when the cure will often be complete." (p. 71.) M. Civiale says:

"In a word, the treatment by soft bougies in ordinary cases is reduced to the daily introduction of bougies whose volume increases from one to three and a haf lines in diameter, and which are so graduated as to cause a regular methodical and progressive dilatation. They are allowed to remain in the canal from two or three minutes to half an hour. The result of this treatment is a progressive diminution of the morbid symptoms, the gradual restoration of the general health, and a complete cure at the end of one or two months." (p. 222.)

Our own experience does not exactly accord with that above stated. We do nót recollect in our own practice that the dilatation treatment is so successful. In most cases we can dilate the canal, and often without experiencing difficulty; but the tendency to relapse is not so easily got rid of, and the necessity for persevering in the occasional use of the bougie, for the remainder of life, has been the common fate of those who have come into our hands with well-developed stricture. It is not indifferent in the treatment of stricture by dilatation, whether the instrument be allowed to remain in the canal one or several minutes or one or several hours; to the one plan the term *temporary*, to the other the term *permanent*, has been applied. The former is the older and more commonly followed plan; the latter is practised by but few. But of these plans there are various modifications. Some persons pass first a bougie which is easily admitted into the stricture; that is immediately withdrawn and a larger one substituted; or each may be allowed to remain one, two, or three hours, or even days, in the canal.

No surgeon is ignorant of the fact that it is possible to dilate the urethra with great rapidity. There is no doubt that when once a passage is obtained for a small bougie, by perseverance one of large size may be passed within thirty-six hours; but it cannot be done without much pain and some danger, and the cure can rarely be thus accomplished. A slower and more rational plan will succeed better, and the patient will be spared much suffering. The treatment by bougies will sometimes excite a good deal of irritation in the canal; it will sometimes cause disorder in the testicles, will sometimes cause false passages, will often fail, that is true; and we are bound to reiterate that, in our experience, unless a stricture be very recent, the treatment by dilatation is only palliative.

The other side of the case is this: that, prudently employed, the pain attendant upon the treatment by *temporary* dilatation is not great, provided it be done gradually and delicately; and there is no doubt that, occasionally, the proper elasticity of the canal is restored and the cure is completed. But when *permanent* dilatation is resorted to, the circumstances are different. The continued presence of a dilating body in the urethra is not usually easily borne; it generally determines heat and pain, which in many cases soon becomes intolerable, and fever is excited; in all cases it irritates the mucous membrane, and determines a more or less considerable discharge; it may cause inflammation of the testicle or the development of abscess. In many cases, when this plan is followed out, the more severe symptoms after a time subside. When this is the effect, the good done at the contracted point soon becomes evident; larger instruments can be passed with scarcely any pain, and the complete dilatation of the canal is then very quickly accomplished; the urine passes freely, and the urethral discharge ceases. Then it is that the patient conceives himself to be cured, and too often his impression is strengthened by his medical attendant; and no further attention to his case is deemed necessary until the stream diminishes and dysury is again present. The fact is, if the dilatation be very quickly effected, the reaction also will quickly follow; for, instead of inducing absorption, as is sometimes the case when the temporary plan is followed, it acts like a wedge. Over and above this—the fact that the *permanent* plan of dilatation is frequently intolerable to the patient and especially uncertain in its results—it condemns the patient to the bed or the sofa, and exposes him to many accidents.

*Cauterization*, as a means of destroying the morbid structure upon which the contraction depends, has been many times exhumed from the oblivion to which it has been again and again consigned. Every new exhumation is made to test the virtues of a new caustic or a new instrument, the principle experiencing no change. The use of the orpiment, the verdigris, and the red precipitate, no one, except Civiale, seeks to revive; nor do many desire to perpetuate the mode of applying them. They were usually incorporated with the extremity of a common bougie. Loyseau, Paré, Wiseman, and John Hunter thought the caustic should be introduced in and protected by a canula; but ultimately their plan was found unsatisfactory, and Hunter caused the nitrate of silver to be inserted into the point of the bougie: such was also the plan of

Home. In all these methods the same principles were sought to be carried out—the destruction of the morbid structure, and the application of the caustic upon its anterior surface. To both those principles there are, we conceive, strong objections. There can be no security against the application of the caustic upon the healthy membrane; and to this it was that the many accidents of cauterization, such as hemorrhage and retention, were owing. The placing of the caustic a little on one side, the reducing it into a paste with mucilage, the previous introduction of a large bougie, or fixing the caustic in the centre of the point of such a bougie, or even the substitution of caustic potash for lunar caustic, do not seem to us to relieve the plan from those inconveniences. Then, supposing the plan to be freed from all these sources of mischief, is it a thing to be desired? We say decidedly, no. If the principle be carried out, the patient's condition would be rendered worse rather than better; and for this reason: it has been abundantly shown that the mucous surface is comparatively unchanged in ordinary cases of stricture; the morbid product is beneath it: now, if we destroy that product with caustic, we must infallibly destroy the mucous membrane before we can get at it, and as certainly the destruction would be followed by an unyielding inelastic cicatrix. The condition of the patient must, in this way, be made worse than before; because no dilating instrument could exercise any efficient influence upon such a structure in making it elastic.

It will be asked, was that the common experience when cauterization was so generally practised? In many instances it was, no doubt. In many more it was not. The reason of this was clearly shown in a former Number of this Review, (July, 1842,) when considering the admirable work of Sir B. Brodie on the 'Diseases of the Urinary Organs.' It is there stated that one of two objects is to be attained by the application of nitrate of silver upon the mucous membrane of the urethra; either a modification of an exaggerated sensibility, or a destruction of substance. With respect to the attainment of the first object it is no longer a matter of doubt. With regard to the second, some observations are necessary. The induration is rarely, if ever, confined to the mucous membrane which, in many cases, appears to have undergone little or no change; it is found to be very much confined to the submucous tissue. Now, if the nitrate be employed for the purpose of effecting the destruction of the indurated mass, it must be evident that before this can be accomplished, there must have been complete destruction of the entire thickness of the mucous coat, there must have been destruction of the submucous hardened tissue, and there must be a subsequent contraction of the surface in the work of cicatrization; in fact, there will be a new contraction, which may require for the remainder of life the daily use of a bougie to overcome. We apprehend, therefore, that the plan of treating stricture by destruction through the agency of nitrate of silver must be abandoned. Although this is the principle upon which that plan of treatment is supposed to be founded, we doubt whether it has often been carried out. We think that the mode in which it has been attempted to carry it out can only very rarely have accomplished the desired object, and for these reasons: the mucous membrane of the urethra is as thick as that which lines the cheeks; now, if a piece of lunar caustic be kept in contact with the latter membrane for a minute, a slough will be formed; in a few hours it will be thrown off, and if in a couple of days afterwards the part be examined, no change of texture will be detected, and many applications upon the same point at short intervals will be required to produce any considerable change of structure. Although, then, we do not deny that the complete destruction of the mucous membrane may be accomplished by the repeated application of lunar caustic, we believe it to be, happily, a rare occurrence.

The experience of this mode of treating stricture was unfavorable, and plans for avoiding some of the objections, and obtaining a greater amount of good were propounded. The first was to take a cast or model of the part, so as to be able to apply the remedy only on the diseased point, and if possible to get it fairly within the stricture. At this Arnott and Ducamp laboured with some success. Mr. Guthrie it appears, like many other surgeons, was also dissatisfied with the effects of caustic applied to the anterior surface of a thick and narrow stricture:

"I was more than *twenty years ago* induced to try the effect of its application to the internal surface, by introducing it into the stricture. The method I adopted was to introduce a hollow silver tube into the stricture with a single eye, which was placed in a narrowed part of the instrument half an inch from its extremity, so that the sort of bulb thus formed on being passed through the stricture, might by catching or drawing it back bring the hole or slit in the narrowed part or neck of it, just opposite the internal surface of the stricture. Into the tube a platina wire was passed, carrying at a proper distance from its extremity a piece of caustic moulded with a hole in its centre for the wire and duly secured, and it looked as if it would act well, but it did not do much. . . . . . In 1825, M. Ducamp, of Paris, dissatisfied with his dilating instrument,. . . published a method by which the argentum nitratum was to be applied to the inside of a stricture also. . . . . . M. Lallemand, not satisfied with the certainty of the application of the caustic by this method, proposed another, which was to introduce into the stricture a hollow elastic bougie having a hole on one side near the end, into which he could pass another which fitted exactly, having affixed in it opposite to the hole existing in the first or hollow instrument a piece of caustic, which might in this manner be safely applied to the inside of the part affected. This method of proceeding I had invented and tried long before M. Lallemand wrote or had practised it. The instruments I had made were shown at my lectures five and twenty years ago; they are still in my possession, but they did not answer my expectations." (pp. 80-2.)

Mr. Guthrie contests the priority as to the mode of applying caustic to the interior of strictures which has so long been accorded to M. Ducamp. At page 80, Mr. Guthrie says it is more than twenty years ago that he was induced to try the effect of the application to the internal surface. This is an indefinite mode of marking time; if we are to take it at twenty years, that will carry us back to 1823, and Ducamp's work was published early in 1822, and not, as Mr. Guthrie states, in 1825. But at page 82, Mr. Guthrie states that he had invented the instrument *twenty-five years ago*, which would carry us back to 1818, three years at least before the publication of Ducamp's work. Let us see whether there be any earlier record of Mr. Guthrie's invention than 1822, because if there be not, with all his merits, the world will be apt to withhold this laurel from his wreath. The earliest record we can find of his invention is the first edition of the work before us, published in 1836. We do not mean to say that this is the earliest recorded intimation of it, but we know no earlier. So far then it is certain that the world will accord to that poor young man who was carried to an early grave, whatever little merit may attach to the invention, and Mr. Guthrie should not grudge it him.

Then, again, Mr. Guthrie had anticipated by many years M. Lallemand, who had improved on Ducamp's instrument. Mr. Guthrie says, and truly, that Lallemand was not satisfied with Ducamp's instrument, and that he proposed another, but where he finds that his improved plan was "to introduce into the stricture a hollow elastic bougie having a hole on one side near the end, into which he could pass another which fitted exactly, having affixed in it, opposite to the hole existing in the first or hollow instrument, a piece of caustic," we cannot tell. It is quite true that Lallemand was dissatisfied with the instrument which Ducamp invented to carry out his plan of applying the caustic to the interior of the stricture, but it is incorrect to say that he used any such instrument as Mr. Guthrie describes for the purpose. In 1825 he published the first part of his 'Observations sur les Maladies des Organes Génito-urinaires,' in which he expresses his opinion in favour of his own modification of Ducamp's instrument. But his instrument, instead of being what Mr. Guthrie describes, is "straight or curved; composed, 1st, of a platina tube open at both ends, destined to protect the nitrate of silver; 2d, of a central part of the same metal, having near its extremity a gutter for the caustic; it should terminate with an olive-shaped enlargement, by which the extremity of the sound should be blocked up; when used the central part is projected several lines beyond the tube." We do not see that this instrument bears any very close resemblance to that which Mr. Guthrie has referred to M. Lallemand. Without stopping longer to point at these trifles in the work before us, we deem it important to offer some observations on cauterization in general.

At present, two opinions prevail with respect to this remedy; one, that the only good which can be obtained from the use of caustic is to modify the sensibility of the part; another, that the obstruction must undergo chemical destruction. The partisans of the former method seek to change the nature of the morbid action, and so facilitate the treatment by bougies; the others conceive that if they can destroy the morbid structure, dilatation will be useless or something worse.

We believe, as we have already stated, that the effects usually anticipated from the use of caustic really do not take place, although many persons appear to consider it as to be placed beyond a doubt they do. There can be no doubt that in some days after the application of lunar caustic upon a stricture the stream of urine will be sensibly enlarged, and this has been regarded as a proof that the canal is enlarged. It is possible that such a result may sometimes be attained; but under such circumstances we have more than once resorted to the following experiment to satisfy ourselves, that when the stream of urine is really enlarged the canal is also enlarged. Before the caustic has been applied, the largest bougie has been passed which the stricture would admit. Eight or nine days have elapsed, and beyond all doubt the stream was sensibly enlarged, but no larger-sized bougie could be passed. We have therefore concluded that in many cases, perhaps in most of those where a decided amelioration has followed the use of caustic, it is owing, not to the destruction of any portion of the morbid structure, but to a modification of the sensibility of the mucous surface at the point. At the same time we believe that this mode of blunting the sensibility at the point, may also facilitate in many cases the passage of a larger instrument than was previously admitted.

There is a singular inconsistency in the reasoning of M. Civiale, in his estimate of the effects of nitrate of silver applied upon the mucous membrane of the urethra. He says (p. 290,) it has generally been admitted, but without reflection, that nitrate of silver acts simply as an escharotic. If it were so, and if each application destroyed, as is said, a portion of the membrane,—its whole thickness should be destroyed when, as is frequently the case, it is applied many times. This is, however, not the case; for when we have an opportunity of examining such a case after death, no traces of the application can be found. At page 295 he says, whenever it is repeatedly and unguardedly applied it occasions serious disorder, such as false passages, &c. Now it seems to us that if it makes false passages it must very completely destroy the whole thickness of the mucous membrane. On the whole, then, every one will be struck by the gloomy pictures painted by the opponents of each. To a certain extent Ducamp also did so, but still he always regarded dilatation as the ultimate means of cure: his followers have lost sight of that, and advocate the destruction of every morsel of morbid product, and hold dilatation to be an unnecessary and painful expedient. The opponents of cauterization point to cases where persons who have been cauterized have had for the remainder of their lives much difficulty in making water; where inequalities, corresponding to cicatrices, have been produced by it; and where obstinate discharges, complaints of the testicle, and other complications have been the result.

That the methodical treatment by bougies is the surest plan of treatment in ordinary cases, there can, we think, be no reasonable doubt; that it is the necessary complement to the treatment by caustic is equally clear; and it was in accordance with this conviction that Ducamp introduced certain modifications in the form of dilating bodies. He conceived that it was desirable to procure the greatest possible amount of dilatation; that is to say, he wished to make the narrowed portion as wide as it was before it became diseased. As the orifice is the narrowest part of the canal, a cylindrical instrument large enough to effect the complete dilatation cannot be passed without much difficulty and pain. Supposing it be deemed proper to introduce such an instrument, either an incision must be made so as to enlarge the orifice, or a bougie, bellied or enlarged at one point, is the only plan by which it can be accomplished. It is found that the orifice may give way for a moment so as to allow the enlarged point to pass without much inconvenience. To the use of the *bellied* bougie, or *bougie à ventre*, Ducamp was accustomed to attach much importance. Mr. Guthrie also thinks the principle an important one. In some cases he "attempts to dilate the diseased part without dilating the whole; without dividing the orifice, to obtain admission for a larger instrument." He says, (p. 74,) "My first attempt in this way was by having a bulb made about an inch from the end of the instrument, which was small at its point and gradually increased to the size desired, from which part it again diminished to a proper-sized shaft;" but as this did not succeed to his wishes he got Mr. Weiss to make a sort of speculum for the purpose. Again: "The opportunity for dilating was in general too tempting to be resisted, and the consequence was that it produced irritation in so many cases that I was forced to give it up." He got a dilator made of softer materials, which was proposed by Dr. Arnott, but which also failed; and he only mentions those circumstances and some improvements he suggested to Dr. Arnott for the purpose of showing the attention he had paid to the improvement of this part of surgery, and that M. Ducamp had no right to lay claim to an invention which Mr. Guthrie states was long known and used in England.

In science the priority is usually conferred on the man who first records an invention, unless good cause to the contrary can be shown. Now, Mr. Guthrie may have used a bellied bougie in 1821, but M. Ducamp first recorded such an invention, not in 1825, as Mr. Guthrie states, but in 1822. And no later in that year than the 6th of May, Deschamps and Percy reported upon it to the Institute. We think, however, that neither M. Ducamp nor Mr. Guthrie need have taken much credit for the invention, for a more utterly worthless instrument for the majority of cases we could scarcely conceive: we are surprised that a surgeon of Mr. Guthrie's experience should have bestowed a moment's attention upon it. M. Civiale, who has had ample opportunities of estimating the value of these instruments, speaks but slightingly of them:

"The bellied bougies which have been proposed to remedy the inconveniences of the cylindrical instrument, are not so useful as has been supposed. Although we may, in certain cases, have recourse to them, they are far from producing the good effects which have been attributed to them. Spite of the eulogiums of Ducamp, who was not always able to avoid exaggeration, nor to preserve impartiality in the parallel between his own plans and those which experience had consecrated, the bellied bougie is in the present day abandoned. The extraordinary dilatation which is sought to be obtained by them is judged to be useless by all practitioners." (p. 208.)

With reference to forced injection, scarification, puncture, incision, and excision, as means of curing stricture, we shall only remark—that the alleged success of the first plan rests on a fallacy, the idea that in cases of stricture the retention of urine is caused by a blocking up of the contracted part by thickened mucus, which can thus be washed away; that the second and third methods, though practised for centuries, should not, we conceive, be used when the contracted point is too small to admit an instrument; and when an instrument can be passed, the puncture or incision is surely not often necessary.

Whatever be the means employed in the treatment of stricture, it is quite certain that the cure is not frequent. Those who rely upon caustic allege that the sound does nothing more than flatten the morbid product; the partisans of dilatation maintain that caustic is followed by an unyielding cicatrix. The true principle of cure we conceive to be this,—to effect the dilatation of the canal with the smallest possible irritation. This principle cannot be carried out by *permanent* dilatation,—the irritation is too great, and the reproduction of the contraction is often rapid; it can rarely follow the repeated use of caustic, which leaves in the end a thicker tissue than it found. We believe that the principle is best carried out by prudent dilatation; the chances of absorption and the regeneration of the primitive elasticity of the canal is then great; but even this treatment may be carried out too energetically,—much irritation may be induced, and the induration increased: and there are cases where the irritability of the surface is so great, that dilatation can only be employed when that irritability has been modified by the application of the nitrate of silver.

